# Characteristics of the oral and gastric microbiome in patients with early-stage intramucosal esophageal squamous cell carcinoma

**DOI:** 10.1186/s12866-024-03233-4

**Published:** 2024-03-15

**Authors:** Han Chen, Xingzhou Jiang, Fengyi Zhu, Ruoyun Yang, Xin Yu, Xiaoying Zhou, Nana Tang

**Affiliations:** 1 Department of Gastroenterology, The First Affiliated Hospital of Nanjing Medical University, 300# Guangzhou Road, Nanjing, 210029 China; 2 The First Clinical Medical College, Nanjing Medical University, Nanjing, China

**Keywords:** Oral microbiome, Gastric microbiome, Early esophageal cancer, Intramucosal esophageal squamous carcinoma, Amplicon sequencing analysis

## Abstract

**Background:**

Oral microbiome dysbacteriosis has been reported to be associated with the pathogenesis of advanced esophageal cancer. However, few studies investigated the potential role of oral and gastric microbiota in early-stage intramucosal esophageal squamous carcinoma (EIESC).

**Method:**

A total of 104 samples were collected from 31 patients with EIESC and 21 healthy controls. The compositions of oral and gastric microbiota were analyzed using 16 S rRNA V3-V4 amplicon sequencing. Linear discriminant analysis effect size (LEfSe) analysis was performed to assess taxonomic differences between groups. The correlation between oral microbiota and clinicopathological factors was evaluated using Spearman correlation analysis. Additionally, co-occurrence networks were established and random forest models were utilized to identify significant microbial biomarkers for distinguishing between the EIESC and control groups.

**Results:**

A total of 292 oral genera and 223 species were identified in both EIESC and healthy controls. Six oral genera were remarkably enriched in EIESC groups, including the genera *Porphyromonas, Shigella, Subdoligranulum, Leptotrichia, Paludibacter*, and *Odoribacter*. LEfSe analysis identified genera *Porphyromonas and Leptotrichia* with LDA scores > 3. In the random forest model, *Porphyromonas endodontalis* ranked the top microbial biomarker to differentiate EIESC from controls. The elimination rate of *Porphyromonas endodontalis* from the oral cavity to the stomach was also dramatically decreased in the EIESC group than controls. In the microbial co-occurrence network, *Porphyromonas endodontalis* was positively correlated with *Prevotella tannerae* and *Prevotella intermedia* and was negatively correlated with *Veillonella dispar.*

**Conclusion:**

Our study potentially indicates that the dysbacteriosis of both the oral and gastric microbiome was associated with EIESC. Larger scale studies and experimental animal models are urgently needed to confirm the possible role of microbial dysbacteriosis in the pathogenesis of EIESC. (Chinese Clinical Trial Registry Center, ChiCTR2200063464, Registered 07 September 2022, https://www.chictr.org.cn/showproj.html?proj=178563)

**Supplementary Information:**

The online version contains supplementary material available at 10.1186/s12866-024-03233-4.

## Introduction

Esophageal cancer is a prevalent form of cancer globally, ranking as the seventh most common type and the sixth leading cause of cancer-related deaths [1]. The two primary histological subtypes of esophageal cancer are squamous cell carcinoma and adenocarcinoma [2, 3]. Squamous cell carcinoma accounts for up to 85% of all esophageal cancer cases [4]. There are notable regional variations in the incidence of the two subtypes. Squamous cell carcinoma is more frequently observed in East, Central, and Western Asia, as well as South Africa [5, 6], whereas adenocarcinoma has a higher prevalence in several developed countries in Northern Europe and North America [6, 7]. The reason for distribution difference is often attributed to the distinct etiology of the two subtypes. The risk factors associated with squamous cell carcinoma include smoking, alcohol consumption, low intake of fruits and vegetables, and consumption of hot beverages [8]. On the other hand, adenocarcinoma is more commonly linked to excess body weight, gastroesophageal reflux disease, and Barrett’s esophagus [9]. In recent years, there has been an increasing focus on the role of digestive tract microecological disorders, in addition to the aforementioned lifestyle habits and pathophysiology, in the incidence of esophageal cancer.

The esophagus, which serves as a connection between the mouth and stomach, is significantly affected by the microbiome of both these regions [10]. Several publications have indicated a potential correlation between oral or gastric microflora and esophageal cancer. A case-control study conducted in China compared the oral microbiota of patients diagnosed with esophageal cancer and healthy volunteers [11]. The study found that patients with esophageal cancer had an enrichment of *Prevotella* and *Veillonellaceae*, as well as a depletion of *Neisseria*. However, it is important to note that the study did not provide any description of the pathological type of esophageal cancer. In a separate study by *Liu et al.* [12], it was reported that the genera *Streptococcus* and *Prevotella* are associated with the prognosis of esophageal squamous cell carcinoma. However, this study did not include a control group for comparison. Peters et al. [13] conducted a nested case-control study in the American population and found a unique composition of the oral microbiome in both esophageal adenocarcinoma and squamous cell carcinoma. They observed the enrichment of *Tanneria forsythiae* and depletion of *Neisseria symbionis* and *Streptococcus pneumoniae* in patients with esophageal adenocarcinoma. On the other hand, patients with esophageal squamous cell carcinoma showed an enrichment of Porphyromonas gingivalis. Another observational study conducted in Iran also revealed that the abundance of *Clostridium difficile* and *erysipelas* in the stomach was associated with esophageal squamous cell dysplasia and carcinoma [14].

Although previous studies have examined the relationship between gastrointestinal flora and advanced esophageal cancer, there is a lack of research specifically focusing on the association of oral microbiota with early-stage esophageal cancer, particularly early-stage intramucosal esophageal squamous cell carcinoma (EIESC). EIESC refers to esophageal squamous cell carcinoma that is confined to the mucosa, with invasion into the surrounding lamina propria and muscularis mucosa, while sparing the submucosa [15]. EIESC often goes unnoticed during white-light endoscopy and can progress to invasive tumors. Currently, the gold standard for diagnosing EIESC is endoscopy with tissue biopsy, which is an invasive procedure [16]. However, esophageal biopsy may sometimes not accurately identify EIESC due to the limited area of cancerous tissue. Consequently, patients may require ongoing endoscopic follow-up, which can lead to a higher social and economic burden. Non-invasive biomarkers for detecting EIESC are still lacking. Additionally, the potential interactions between the microbiome in different parts of the gastrointestinal tract in relation to esophageal cancer are not fully understood. Therefore, this study aims to investigate the correlation between early esophageal squamous cell carcinoma and the oral and stomach microflora, as well as explore the interactions between these two microbiomes. The study also aims to identify potential microbial biomarkers for predicting early esophageal squamous cell carcinoma.

## Method

### Study design

A case-control study was performed in the First Affiliated Hospital of Nanjing Medical University, a tertiary care university medical center. Patients pathologically diagnosed with EIESC after receiving endoscopic submucosal dissection (ESD) were consecutively enrolled between September 2022 and January 2023. Inclusion criteria were: (1) histopathological diagnosis of EIESC on ESD specimens; (2) pT1 stage carcinoma (no tumor invasion beyond the mucous layer); (3) no history of previous malignancies and anticancer therapies; (4) patients who agreed to provide saliva and gastric biopsy samples for the study. Exclusion criteria: (1) pathological diagnosis of adenocarcinoma or other histopathological types; (2) mixed type of esophageal cancer; (3) tumor with the undefined pathological origin and metastatic cancer; (4) patients younger than 18 years; (5) a history of infection and antibiotics or probiotics use during the past four weeks before admission; (6) current or previous history of *Helicobacter pylori* infection confirmed by the carbon urea breath test; (7) previous medical history of hematologic or rheumatic autoimmune disease; (8) acute or chronic infections during hospitalization; (9) a previous aspirin or warfarin-taken history. Written informed consent was obtained from each enrolled patient for collecting the saliva and gastric biopsy samples and obtaining relevant medical information during hospitalization. The control group consisted of individuals who underwent upper gastrointestinal endoscopy at the outpatient center between September 2022 and January 2023. No obvious abnormalities were observed under endoscopy in these individuals. They voluntarily provided saliva and gastric biopsy samples for the study. Exclusion criteria for the control group included: (1) presence of endoscopically-reported lesions such as atrophic gastritis, reflux esophagitis, upper gastrointestinal tumors, dysplasia, ulcers, erosion, and submucosal lesions; (2) individuals who refused to provide saliva and gastric biopsy samples;(3) individuals under 18 years of age; (4) history of infection and use of antibiotics or probiotics in the four weeks prior to admission; (5) current or previous history of Helicobacter pylori infection confirmed by the carbon urea breath test; (6) previous medical history of atrophic gastric, inflammatory bowel diseases, hematologic or rheumatic autoimmune disease; (7) acute or chronic infections; (8) previous use of aspirin or warfarin.

The 8th edition AJCC/UICC staging system of esophageal cancer was applied [17]. Tumor sizes were determined as the maximum diameter in two dimensions, measured by Vernier calipers. Histologic grade was categorized as well-differentiated (G1), moderately differentiated (G2) and poorly differentiated (G3). Macroscopic tumor type was classified using the 2016 Japanese Classification of Esophageal Cancer, 11th.

Edition [18]. MedCalc (Version 20.100t) was used to calculate the sample size. The parameters were set as follows: the ROC curve (AUC) at the 0.05 alpha level, the 0.1 beta level (power is 90%), the expected AUC was 0.8, and the null hypothesis value was set to 0.5. The minimum sample size required for each group was 17. The study was approved by the Institutional Ethics Committee of the First Affiliated Hospital of Nanjing Medical University and was then registered in the Chinese Clinical Trial Registry center (www.chictr.org.cn; registration no. ChiCTR2200063464).

## Saliva sample collection

The saliva samples were collected from eligible patients using the Navazesh method [19]. Prior to the collection procedure, patients were instructed to rinse their mouths thoroughly with deionized water 30 min beforehand. They were then asked to sit comfortably for 5 min and minimize orofacial movements. The saliva (2 ml) was collected from the bottom of the mouth and patients were instructed to spit it into a sterile saliva collection tube (43,805, Shanghai Xiyan Technology Co., Ltd.) every 60 s. Each sample was mixed thoroughly and centrifuged (3000 g at 4℃) for 10 min. The supernatant (500ul) was extracted into a sterile freezing vial (V9255, Germany Merck Group Co., Ltd.) and immediately transferred to liquid nitrogen. Finally, the samples were stored in a refrigerator at -80℃.

### Gastric biopsy sample collection

Gastric biopsy tissues were collected from each patient during the esophageal ESD procedure. Prior to the invasive steps of ESD, which included marking and submucosal injection, two pieces of biopsies were obtained from the gastric antrum using disposable sterile endoscopic biopsy forceps (MTN-BF-23/16-A-C, Nanjing Micro-Tech Co., Ltd). The collected tissues were then placed into sterile freezing vials (V9255, Germany Merck Group Co., Ltd.) and immediately transferred to liquid nitrogen for storage in a refrigerator at -80 ℃. The biopsy tissues were disrupted by bead-beating after digestion with a mutanolysin and lysozyme enzyme cocktail (Sigma), according to cetyltrimethylammonium bromide (CTAB) based protocol [20].

### DNA extraction and 16 S rRNA gene sequencing

Microbial genomic DNA was extracted using the CTAB Method and stored at -20 °C. The V3-V4 hypervariable regions, approximately 468 bp in length, were amplified by polymerase chain reaction (Primers: 338 F (5′-ACTCCTACGGGAGGCAGCA-3′) and 806R (5′-GGACTACHVGGGTWTCTAAT-3′). PCR samples were quantified using the Quant-it PicoGreen dsDNA Assay Kit on a Microplate reader (BioTek, FLx800) and mixed based on the amount of data for each sample. DNA library construction was performed using the TruSeq Nano DNA LT Library Prep Kit (Illumina Scientific Co., Ltd). The quantity and quality of each library were measured using the Quant-iT PicoGreen dsDNA Assay Kit and Agilent High Sensitivity DNA Kit, respectively. For qualified libraries, the NovaSeq 6000 SP Reagent Kit (500 cycles) on the Illumina NovaSeq machine was used for 2 × 250 bp double-ended sequencing. Raw data from the 16s rRNA sequence was processed by Personalbio Technology Co., Ltd. (Shanghai, China). Amplicon sequence variants (ASVs) were integrated into merged taxonomic abundance and taxonomy classification tables. All amplicon raw data have been submitted to the Sequence Read Archive (SRA) in NCBI (Archive number: PRJNA961904).

### Statistical analysis

Statistical analysis was conducted using R software (version 4.1.0, http://www.Rproject.org/). Normality test were applied by Shapiro-Wilk and Kolmogorov-Smirnov test. Data with normal distribution were considered if the p-value is less than 0.05 and is presented as mean and Standard Deviation, and data with non-normal distribution were presented as median with Interquartile Range (Q). Wilcoxon Mann‒Whitney test was performed for unpaired data with a non-normal distribution, while the Wilcoxon signed-rank test was performed for paired data. Pearson chi-square test was used to compare categorical variables with no more than 20% of cells with expected frequencies < 5, and Fisher’s exact test was used if > 20% of expected cell counts are less than 5.

### Analysis of microbial diversity

The Chao1, Shannon, and Simpson indices were applied to evaluate the alpha diversity of microbial communities using QIIME2 (2019.4) platform (https://docs.qiime2.org/2019.4/) and ggplot2 R package (https://cran.r-project.org/web/packages/ggplot2/). The alpha-rarefaction curve was generated using the QIIME2 (2019.4) platform (https://docs.qiime2.org/2019.4/). Beta-diversity was visualized using the unconstrained principal coordinate analyses (PCoA) scatter plots via calculating Bray-Curtis distances. An upset plot was using UpSetR package (https://cran.r-project.org/web/packages/UpSetR/) to identify unique and common OTUs.

### Differential analysis of microbial compositions

The pairwise differential analysis was performed using the Mann-Whitney U test. Multiple comparisons were performed using the Kruskal-Wallis test. The volcano plot was generated using the ggrepel and ggplot2 R packages (https://cran.r-project.org/web/packages/ggplot2/). The manhattan plot was generated using metagenomeSeq and ggplot2 packages. Linear discriminant analysis effect size (LEfSe) was calculated with the online website (http://huttenhower.sph.harvard.edu/galaxy) to determine significant biomarkers for differentiating CRC and control samples [21]. The cutoff value was defined if the linear discriminant analysis (LDA) score was more than 2.0 and *P* < 0.05. The random forest model [22] was built using the randomForest and pROC package. The predictive performance was optimized by selecting species that displayed the best discriminatory power.

### Microbial interaction with clinicopathological factors

The Spearman correlation analysis was performed and visualized in R using the package corrplot, ggcor, ggplot2, and pheatmap packages. Mantel analysis was also performed to investigate the relationship of significantly different microbes/metabolites with clinical characteristics of mice, using the R packages of LinkET [23].

### Co‑occurrence network

A correlation matrix was developed by calculating the pairwise Spearman’s rank correlations in all EIESC saliva or gastric biopsy samples. A correlation between two microbes was considered statistically robust if Spearman’s correlation coefficient was > 0.6 and the p-value was < 0.01 [24]. To reduce the chances of obtaining false-positive results, the p-values were adjusted using the Benjamini–Hochberg method. The molecular ecological network analyses (MENA) were applied to construct random matrix theory (RMT) based on co-occurrence bacterial networks and was visualized in Cytoscape Version 3.9.1. The most densely connected node was defined as the hub microbe, and hub microbes were searched using the cytohubba module in Cytoscape Version 3.9.1.

### The prediction of microbial taxa functional pathways

PICRUSt2 (https://huttenhower.sph.harvard.edu/picrust/) was used for the prediction of amplicon functions. Bray-Curtis distances were applied to evaluate the similarity of data from the microbial composition [25]. Kyoto Encyclopedia of Genes and Genomes (KEGG) functional prediction was performed. The Benjamini-Hochberg procedure was applied to correct the false discovery rate due to multiple tests.

## Results

### The baseline characteristics of the enrolled samples

**Fig. 1:**
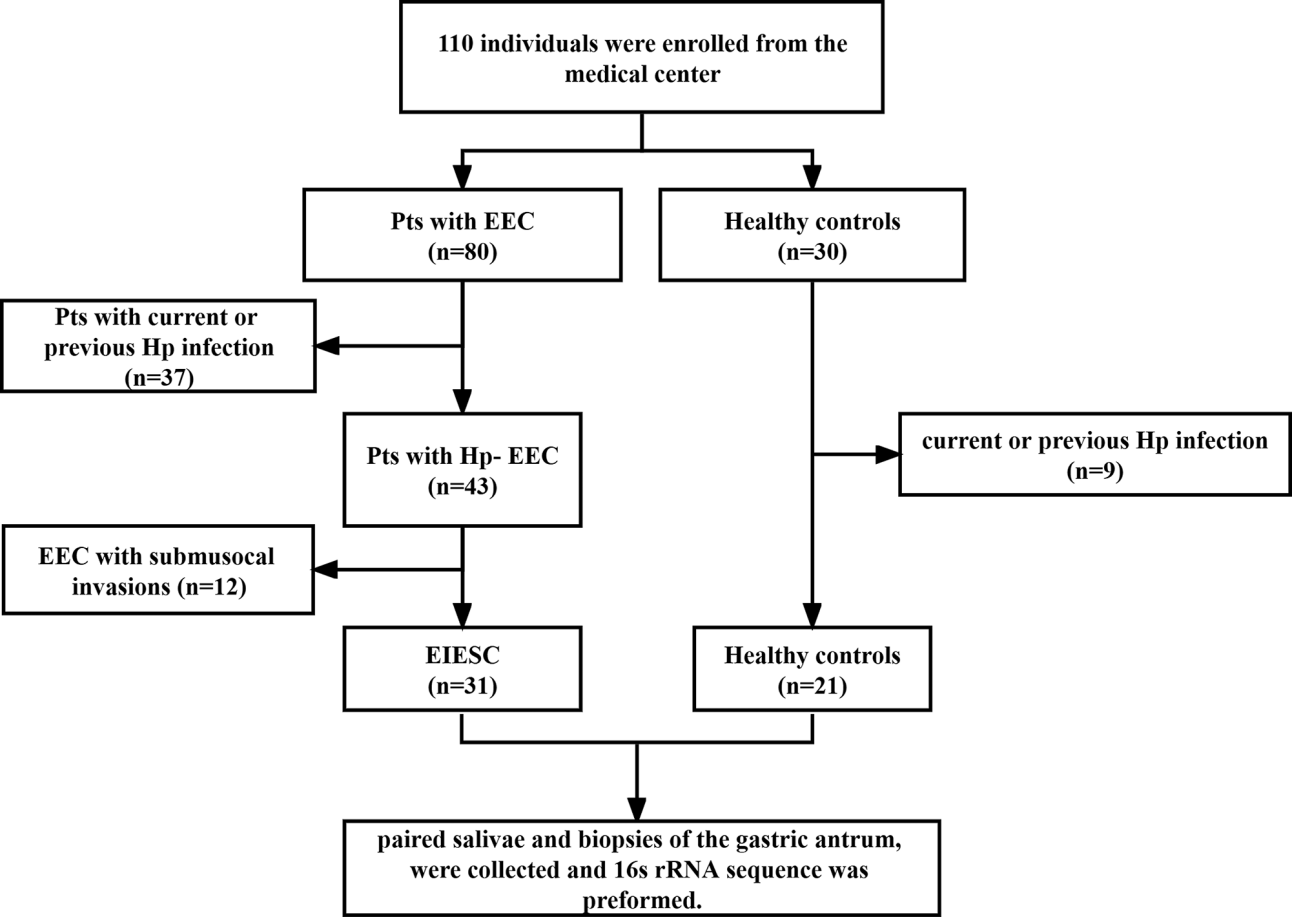
The flowchart of the study

Figure 1 presents the flowchart of the study. A total of 110 individuals were enrolled. After following the inclusion and exclusion criteria, 52 patients (31 EIESC and 21 controls) were finally included. A total of 104 samples, including 52 paired saliva and biopsies of the gastric antrum, were collected. The baseline characteristics were shown in Table 1. There were no significant differences in age, sex, and history of smoking or drinking between the EIESC group and healthy controls (all *p* > 0.05).

**Table 1:** Baseline characteristics of patients in EIESC and control groups

No. of patients	EIESC (*n* = 31)	Controls (*n* = 21)	p
**Age** (median [IQR])	70 [60, 71]	64 [59, 69]	*0.094*
**BMI** (median [IQR])	21.61 [21.40, 22.03]	22.31 [21.10, 22.55]	*0.309*
**Sex** (n, %)			*0.523*
Female	12 (38.7)	10 (47.6)	
Male	19 (61.3)	11 (52.4)	
**History of smoking** (n, %)			*0.494*
Yes	9 (29.0)	8 (38.1)	
No	22 (71.0)	13 (61.9)	
**History of drinking** (n, %)			*0.624*
Yes	7 (22.6)	6 (28.6)	
No	24 (77.4)	15 (71.4)	
**Family history of Esophageal cancer** (n, %)			*0.985*
Yes	3 (9.7)	2 (9.5)	
No	28 (90.3)	19 (90.5)	

Note ^†^*p* value was derived from the Mann-Whitney test in data of continuous variables with abnormal distribution (M, Median; IQR, Interquartile Range). *p* value was derived from the Chi-square test or fisher’s exact test in data of categorical variables from EIESC and controls (n,%). Abbreviation: EIESC: early-stage intramucosal esophageal squamous cell carcinoma; BMI: body weight index

### No significant difference in alpha diversity between EIESC and healthy groups

**Fig. 2:**
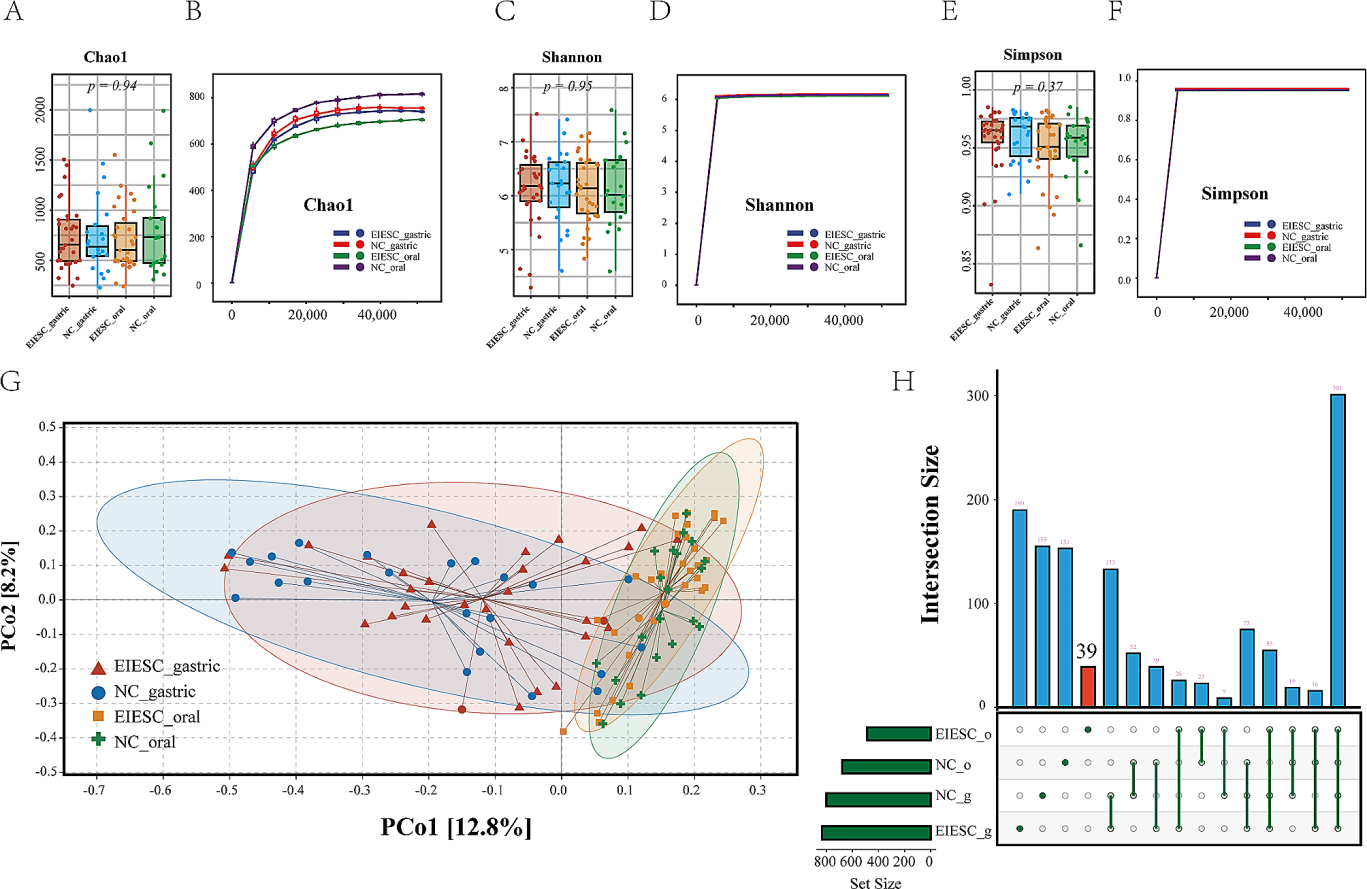
Comparisons of microbial diversity. **A, C, and E**: the boxplot of alpha diversity between the EIESC and controls using the Chao1 (**A**), Shannon(**C**), and Simpson index (**E**). **B, D and F**: the Rarefaction curves of the diversity detected compared with the predicted diversity. The x-axis represents the number of sequences sampled while the y-axis represents the estimated Chao1 (**B**), Shannon index (**D**), and Simpson index (**F**). **G**: PCoAs of Bray‒Curtis distances on the microbiota distributions. Each dot represents a patient with EIESC or controls. Points clustered in red and blue represent the gastric microbial composition of the EIESC and controls, whereas the points clustered in yellow, blue represent the oral microbial composition of the EIESC and controls. EIESC: early-stage intramucosal esophageal squamous cell carcinoma; PCoAs: principal coordinate analyses. **H**: UpSet plot of differently-distributed taxa. The left graph represents the total number of differently-distributed species (X-axis) in EEC saliva (EEC_o), Control saliva (NC_o), EEC gastric biopsy (EEC_g), and control gastric biopsy groups (EEC_g) (Y-axis). The right graph represents the intersection of sets of species in multiple groups. Each column corresponds to a group or set of groups (dots connected by lines below the X-axis) containing the same species. The number of species in each set appears above the column, while groups shared are indicated in the graphic below the column. *, **, *** stands for p-value < 0.01, 0.005 and 0.001, respectively). EEC: early esophageal cancer

16 S rRNA gene sequencing of the V3-V4 region generated a total of 28,337,336 reads (with a median of 280,529 reads per sample and a range of 187,908 − 299,982 reads per sample). All samples met the sequencing quality criteria, with Q30 scores exceeding 90% and average good’s coverage estimates surpassing 99% (Table S1). Alpha diversity (Fig. 2A-F) showed no significant difference between EIESC and healthy groups in both the saliva and gastric biopsy samples, according to the Chao1, Shannon, and Simpson indices (all *p* > 0.05). Refraction curves of Chao1, Shannon, and Simpson tend to flatten out, indicating the sequencing detecting depth has reached saturation.

### Heterogeneous beta diversities between saliva and gastric samples

When evaluating the beta diversity, there were no significant heterogeneous microbiota distributions of each saliva and gastric sample between the EIESC and control groups, respectively. However, the difference in beta diversity was observed in PcoA Axis1 between the saliva and gastric samples in each EIESC group (*p* < 0.001) and control group (*p* < 0.001), respectively (Fig. 2G).

### Different microbiome compositions between EIESC and controls

**Fig. 3:**
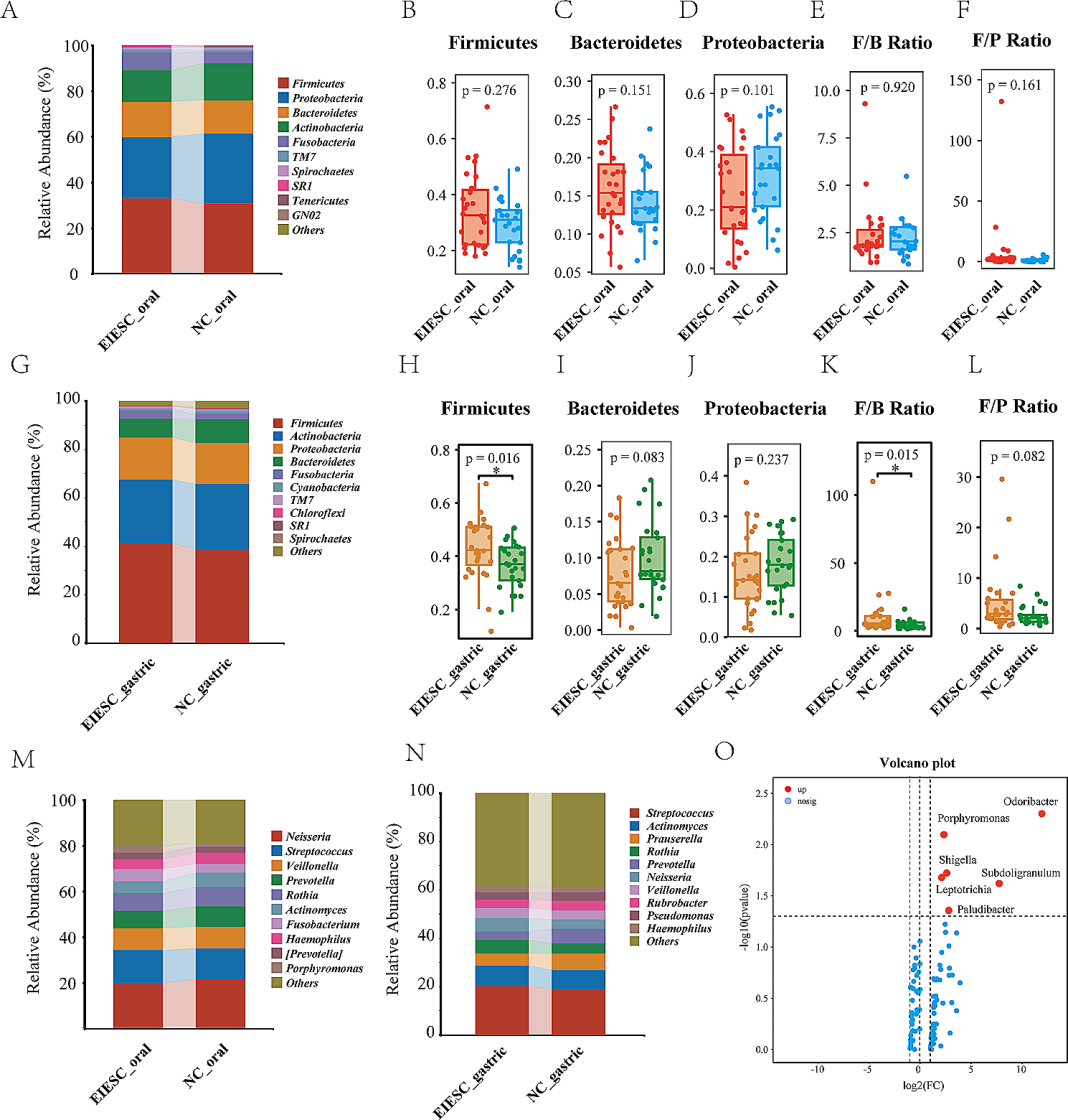
The differential analysis of microbiota compositions between EIESC and controls at the phylum and genus level. A: the vertical bar chart presenting the oral microbiota compositions between EIESC and controls at the phylum level. The x-axis represents each sample and its group, and the y-axis represents the relative abundance. **B-F**: Boxplots showing the relative abundance of Firmicutes, Bacteroidetes, Proteobacteria, Firmicutes/Bacteroidetes (F/B) ratio, and Firmicutes/Proteobacteria (F/P) ratio in saliva samples. **G**: the vertical bar chart presenting the gastric microbiota compositions between EIESC and controls at the phylum level. **H-L**: Boxplots showing the relative abundance of Firmicutes, Bacteroidetes, Proteobacteria, F/B ratio, and F/P ratio in gastric biopsy samples. **M-N**: the vertical bar chart presenting the oral (**M**) and gastric (**N**) microbiota compositions between EIESC and controls at the phylum level. **O**: Volcano plot: the log2 fold-change indicates the mean relative abundance for each taxon. Each dot represents one genus. The blue dots represent no significant expression difference between the MHO and control groups, the red dots represent EIESC-enriched genus

A total of 292 oral genera and 223 oral species (with unidentified species excluded) were identified in both EIESC and healthy controls. Thirty-nine microbial species were distinctively identified in EIESC groups (Fig. 2H, Table S2). At the phylum level, there was no significant difference in the abundance of *Firmicutes* (Mean Relative Abundance (MRA), EIESC versus Control: 32.6% versus 31.1%, *p* = 0.276), *Bacteroidetes* (MRA 15.4% versus 13.4%, *p* = 0.151), and *Proteobacteria* (MRA 20.9% versus 34.3%, *p* = 0.101) between EIESC and controls (Fig. 3A-D). The *Firmicute/Bacteroidetes* ratio (F/B Ratio) (1.87 versus 2.04, *p* = 0.920) and *Firmicute/Proteobacteria* ratio (F/P Ratio) (1.48 versus 0.96, *p* = 0.161) also showed no significant difference between the two groups (Fig. 3E-F). As for the gastric microbial composition, the abundance of *Firmicutes* (MRA 42.3% versus 37.2%, *p* = 0.016) and F/B Ratio (5.46 versus 3.84, *p* = 0.015) were significantly increased in patients with EIESC when compared with healthy controls, whereas the abundance of *Bacteroidetes* (MRA 6.5% versus 8.0%, *p* = 0.080), and *Proteobacteria* (MRA 14.2% versus 18.4%, *p* = 0.237) and F/P Ratio (2.9 versus 2.1, *p* = 0.080) show no statistical difference (Fig. 3G-L).

At the genus level, the vertical bar chart presenting the top 10 abundant genera of the oral and gastric microbiota compositions between EIESC and healthy controls at the genus level were shown in Fig. 3M-N. Six oral genera were remarkably enriched in EIESC groups (Fig. 3O, Table S3), including the genera *Porphyromonas* (MRA 3.19% versus 1.37%, *p* = 0.008), *Subdoligranulum* (MRA 0.002% versus 0.0002%, *p* = 0.024), *Leptotrichia* (MRA 2.96% versus 1.40%, *p* = 0.021), *Paludibacter* (MRA 0.12% versus 0.04%, *p* = 0.044), *Shigella* (MRA 0.02% versus 0.008%, *p* = 0.019), *and Odoribacter* (MRA 0.007% versus 0.0006%, *p* = 0.005).

**Fig. 4:**
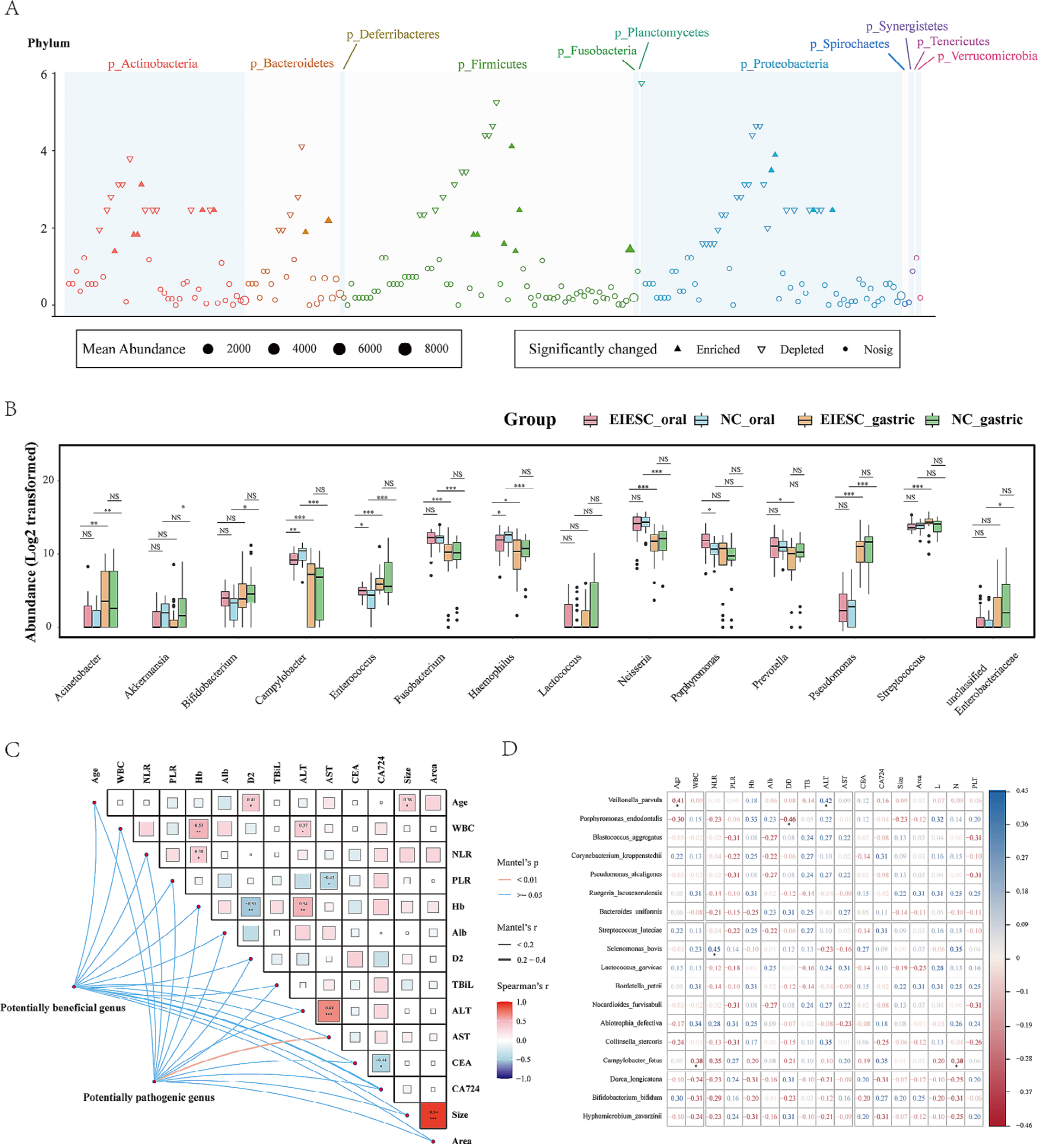
The differential analysis of microbiota compositions between EIESC and controls at the species level and microbial correlation with clinicopathological factors. **A**: Manhattan plots showing the distributions of each oral species identified in EIESC and individuals. Significantly-enriched species are depicted as transparent triangles, significantly-depleted species are presented as inverted solid triangles, and species with no statistical significance are depicted as full circles. The color of each dot represents the different phylum affiliations, and the size stands for their relative abundance. The light-green and light-blue boxes are used to denote different phylum groups. **B**: boxplots showing the abundance (Log 2 transformed) of typical genera in four different groups. The Wilcoxon Test was performed and *, **, *** stands for p-value < 0.01, 0.005 and 0.001, respectively). **C**: Visualization of the Mantel test. The triangle on the right side represents pairwise comparisons of clinically relevant factors with a color gradient denoting Spearman correlation coefficients. The potentially beneficial genus and potentially harmful genus were related to each clinical factor, respectively, using partial (geographic distance– corrected) Mantel tests. Edge width corresponds to the Mantel r statistic for the corresponding distance correlations, and edge color denotes the statistical p significance value. **D**: Heatmap matrix plot of Spearman’s correlation coefficients (ρ) among significantly enriched or delpeted species. The absolute value of ρ is indicated by a color code explained in the legend. The blue color indicates a positive correlation, whereas red represents a negative one. The scale of a square is proportional to ρ2. Cells above the matrix diagonal refer to specific ρ values and their statistical significance (p-value). Significance levels *p* < 0.05, *p* < 0.01, and *p* < 0.001 are indicated by *, **, and ***, respectively, whereas *p* > 0.05 is presented p explicitly

At the species levels, the different compositions of oral species was shown in Fig. 4A. A total of 19 species were significantly enriched in the EIESC group, whereas 49 species were depleted when compared with the healthy controls (Fig. 4A, Table S4).

To further investigate the composition difference, we specifically compared the abundance change of the common bacterial genera in different groups (Fig. 4B). The abundance of *Bifidobacterium* presented no significant change in both oral and gastric microbiome between the EIESC and control groups. The gastric genera *Akkermansia* was significantly depleted in patients with EIESC compared with control groups. There was a remarkable enrichment of *Bifidobacterium* in gastric microbiome when compared with the oral genera in healthy controls, whereas this tendency of enrichment was not presented in the EIESC group. We also observed a remarkably decreased abundance of the genera *Campylobacter, Fusobacterium, Haemophilus*, and *Neisseria* in the gastric microbiome compared with the oral microbiome in both the EIESC and control groups, indicating the possible elimination of these genera with gastric acid. However, the abundance of the genera *Acinetobacter, Enterococcus*, and *Pseudomonas* was significantly increased in the gastric environment compared with oral microbiota. In patients with EIESC, the abundance of *Streptococcus* in the stomach was remarkably enriched compared to that in the oral cavity, but this trend was not observed in healthy controls.

### No significant correlation of oral microbiome with clinical factors in EIESC

To track the association of significantly changed oral taxa with clinicopathological characteristics in EIESC groups, we applied Mantel (Fig. 4C) and Spearmen correlation tests (Fig. 4D). No significant correlations were observed of oral species with tumor size, tumor area (pathological ESD specimens), and common serum blood tests such as WBC, Alb, and Hemoglobin (all rho < 0.4, *p* > 0.05). The increased abundance of potentially pathogenic genera was positively correlated with the AST levels in Mantel’s correlation test.

### The LefSe and RF model identified several oral microbial biomarkers for EIESC

**Fig. 5:**
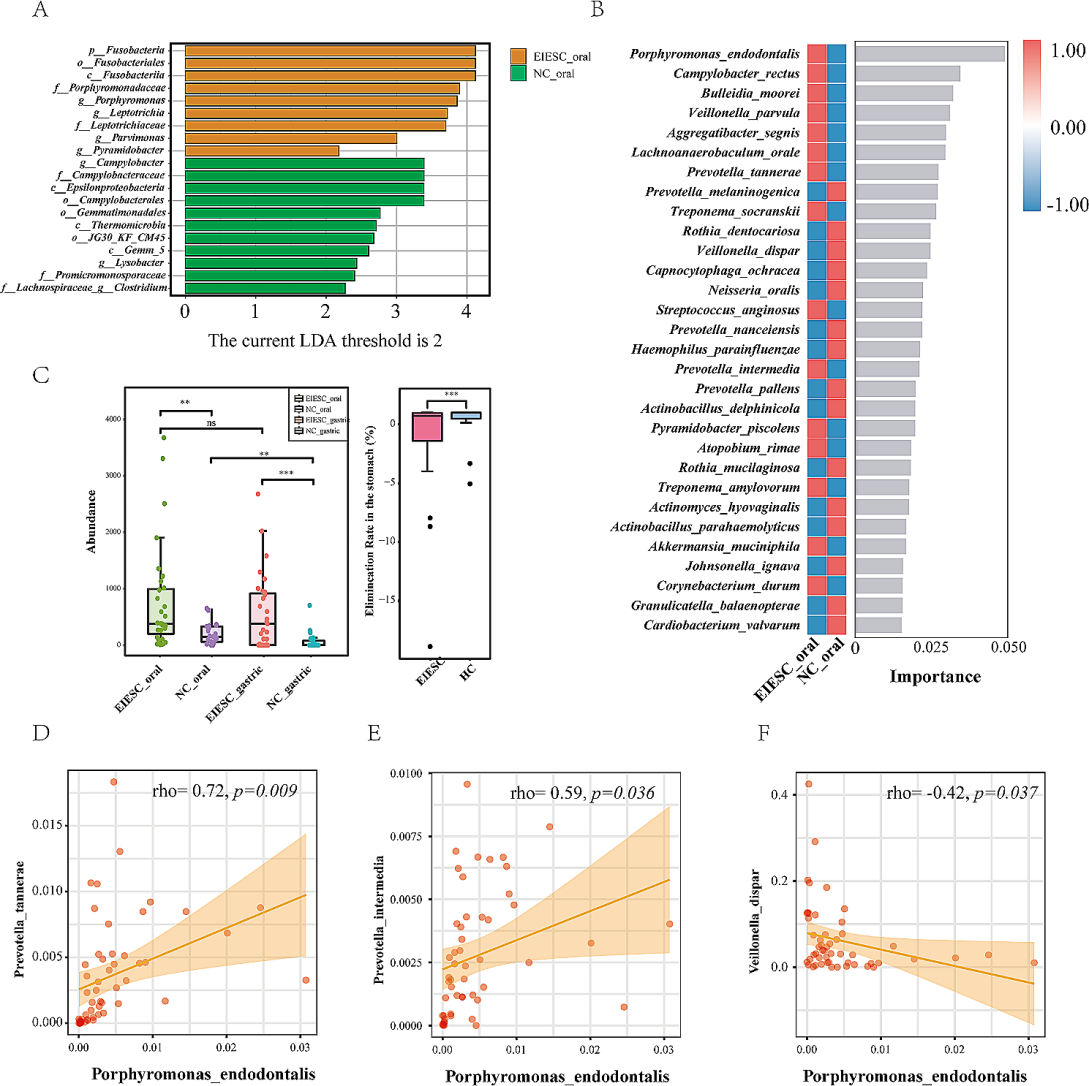
Identification of the oral microbial biomarkers. **A**: LEfSe analysis. Plot of LDA Effect Size. The length of the bar column represents the LDA score. The figure shows the oral microbial taxa with significant differences between the EIESC (orange) and Control (green) (LDA score > 2). **B**: Random Forest model of the representative 30 microbial biomarkers to predict EIESC based on their mean decrease scores of the optimal model performance. **C**: boxplots showing the different abundance of Porphyromonas endodontalis in four different groups (Left boxplot), and the elimination rate of *Porphyromonas endodontalis* from the oral cavity to the stomach (Right boxplot). Significance levels *p* < 0.05, *p* < 0.01, and *p* < 0.001 are indicated by *, **, and ***, respectively, whereas *p* > 0.05 is presented p explicitly. D-F: Correlation plot of *Porphyromonas endodontalis* with strong correlation with three species, including *Prevotella tannerae* (D), *Prevotella intermedia* (E) and *Veillonella_dispar* (F)

LefSe analysis identified two genera with LDA score > 3, including the genera *Porphyromonas* and *Leptotrichia* (Fig. 5A). In the random forest model, we identified that the oral species of *Porphyromonas endodontalis, Campylobacter rectus*, and *Bulleidia moorei* were the top 3 ranked species to differentiate EIESC with healthy controls (Fig. 5B). Next, we specifically investigated the abundance of *Porphyromonas endodontalis* in different microbial samples in both the EIESC and controls. Interestingly, we observed a significant enrichment of oral *Porphyromonas endodontalis* in patients with EIESC compared with healthy controls. The elimination rate of *Porphyromonas endodontalis* from the oral cavity to the stomach was also dramatically decreased in the EIESC group than controls (Fig. 5C). Besides, we also observed the strong positive correlation of *Porphyromonas endodontalis* with two *Prevotella* species, including *Prevotella tannerae, and Prevotella intermedia* (Fig. 5D-E), whereas the abundance of *Veillonella_dispar* was negatively correlated with that of *Porphyromonas endodontalis* (Fig. 5F).

### The co-occurrence network identified hub species of EIESC patients

**Fig. 6:**
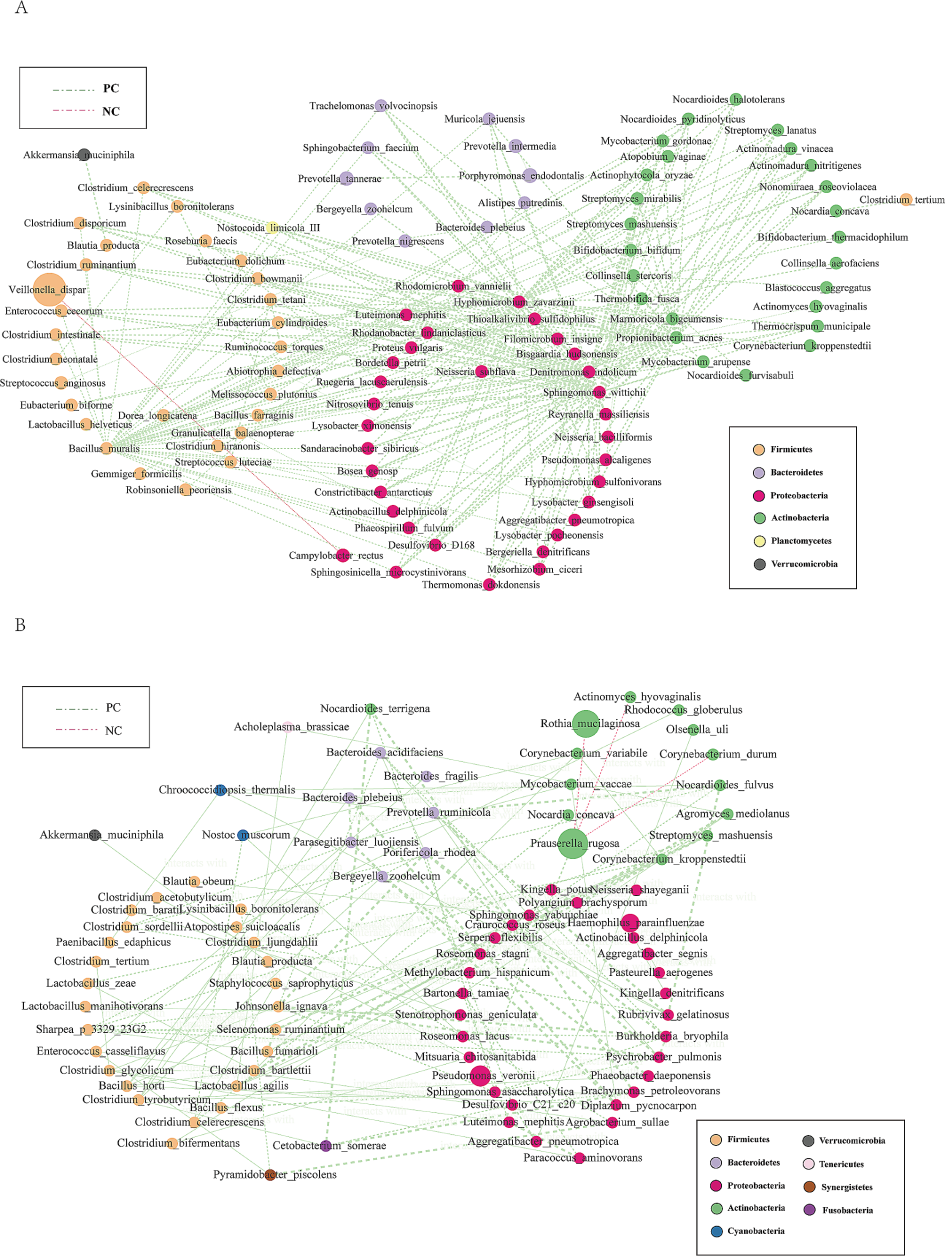
Co-occurrence network visualization of the oral (**A**) and gastric (**B**) microbial interactions in the EIESC individuals. The lines connecting nodes (edges) represent a positive (light green) or negative (red) co-occurrence relationship. The color of each dot represents the different taxonomic affiliations of the species (phylum level), the width of the edges reflects the absolute value of correlation coefficients, and the size corresponds to their relative abundance

The co-occurrence patterns among the EIESC patients were explored using network inference based on strong (|ρ|> 0.6) and significant (*p* < 0.01) correlations. The oral microbiome network (Fig. 6A, Table S5) was composed of 99 nodes (microbes) and 250 edges. The top 3 ranked hub microbes were *Bacillus muralis, Denitromonas indolicum*, and *Sphingomonas wittichii* using the maximal clique centrality (MCC) method within the cytohubba. The gastric microbiome network (Fig. 6B, Table S6) was composed of 88 nodes (microbes) and 115 edges. The top 3 ranked hub microbes were *Johnsonella ignava, Burkholderia bryophila*, and *Porifericola rhodea.* (Fig. 6B). Using the PICRUSt2 method, no significant KEGG pathways were strongly associated with EIESC (Table S7).

## Discussion

Our findings highlight the potential role of the oral microbiome in the development of EIESC. In general, we observed that patients with EIESC had distinguished oral microbial compositions compared with healthy controls. Although we observed no significant difference in microbial diversities, our analysis revealed distinct distributions of several microbial taxa, such as the genera *Porphyromonas* and *Leptotrichia*, between the EIESC and control groups.

*Firmicutes, Bacteroidetes, and Proteobacteria* are the three major bacterial species most commonly found in the human gut microbiota [26, 27]. The F/B ratio is considered a potential biomarker of gut dysbiosis [27], which has been reported in several diseases [28, 29]. Different tumor samples were found to have different F/P ratios [30]. In our study, no statistical differences were observed in the F/P and F/B ratios of the oral microbiome. However, the F/B ratio of the EIESC group was significantly lower than that of the normal growth group, indicating the potential gastric dysbiosis in patients with EIESC.

*Porphyromonas*, particularly *Porphyromonas gingivalis*, has been consistently found to have a higher abundance in saliva samples [13], blood serum [31], and tumor tissues [32] of patients diagnosed with esophageal squamous cell carcinoma. This genus has been potentially linked to an increased risk [13, 33] and poor prognosis [31, 32] of esophageal squamous cell carcinoma. Our findings suggest that the abundance change of genus *Porphyromonas* may occur even at an early stage of esophageal cancer. The genus *Porphyromonas* is a potentially harmful bacterium linked to periodontitis [33]. Several previous studies have demonstrated a link between inadequate oral hygiene and a higher likelihood of developing esophageal cancer [34, 35]. In our study, we observed a higher presence of the *Porphyromonasz* genus in the saliva of EIESC patients. Therefore, our findings indicate that the *Porphyromonas* genus could potentially contribute to the increased vulnerability to esophageal cancer in individuals with periodontal diseases. However, different from previous studies [31, 32], our findings revealed a significant presence of *Porphyromonas endodontalis, another Porphyromonas spp.*, in the EIESC groups. Similar to *Porphyromonas gingivalis, Porphyromonas endodontalis* commonly populates dental plaque and is associated with endodontic infections and abscesses [36]. However, there have been no previous report of a significant change in the abundance of *Porphyromonas endodontalis* in esophageal cancer. Our findings suggest that *Porphyromonas endodontalis* could potentially serve as a microbial biomarker to distinguish EIESC from controls. Besides, our study revealed strong positive correlations between *Porphyromonas endodontalis* and two *Prevotella* species: *Prevotella tannerae* and *Prevotella intermedia*. These two species were also identified as important biomarkers in the random forest model of EIESC. It is worth noting that *Prevotella tannerae* and *Prevotella intermedia* have been associated with periodontal and endodontic infections [37], indicating a potential link between oral hygiene and the progression of esophageal cancer at an early stage.

Interestingly, we observed that the microbial composition of esophageal cancer varied depending on the histopathology. Peters et al. [13] conducted a study on the oral microbiota of the two main types of esophageal cancer and found that esophageal adenocarcinoma showed significant changes in genera compared to squamous cell carcinoma. They identified no significant microbiota-associated functional pathways in patients with advanced squamous cell carcinoma. Similarly, in our study, we also found no significant differences in functional pathway predictions between EIESC and the control group. This suggests that the oral microbiome may potentially have a lesser role in the development of esophageal squamous cell carcinoma compared to adenocarcinoma. In fact, other risk factors such as smoking, alcohol consumption, and consumption of pickled food may have a greater impact on patients with EIESC. Therefore, future cancer screening strategies should consider integrating various factors and establishing comprehensive predictive models that incorporate microbial biomarkers and other esophageal cancer risk factors.

In addition, we also investigated the characteristics of gastric microbiota in each patient paired with their saliva samples, which has not been studied in previous studies. There were significant microbial diversity and composition differences between the oral and gastric mucosa in both the EIESC and controls. We observed a notable decrease in the abundance of several gastric genera due to the elimination of gastric acid, while some genera, such as *Acinetobacter*, *Enterococcus*, and Pseudomonas, were even more abundant in the stomach compared to the oral cavity. In EIESC patients, we observed a decreased abundance of *Streptococcus* in the stomach compared to that in the oral cavity, and the gastric *Bifidobacteria* was significantly depleted. These results all indicate that there is also an imbalance in the gastric microbiome in EIESC patients.

Our study has several limitations. First, it is a case-control study with a relatively small sample size. The main reasons for a relatively small sample size include: (1) we excluded all patients with previous and current H. pylori infection as an important confounding factor that may affect the gut microbiome; (2) we only include patients with intramucosal cancer which represents a very early stage of esophageal cancer. The sample size in control group was also very small. It is important to note that the microbiome obtained from these controls might not necessarily represent a healthy microbiome. This is because the microbiome of a healthy person might not yet fully understood and each individual has a highly diverse microbiome [38–41]. Second, we did not investigate the periodontal status of participants to study whether all the oral species we identified were independent of periodontal disease. Third, we only applied the 16s rRNA sequence which had limitations to detect significant taxon at the species level. Thus, further research is warranted to investigate the role of these species in the pathogenesis of esophageal cancer using a larger scale of sample size with more accurate microbial analysis, such as metagenomic analysis.

In conclusion, this study potentially indicates that the dysbacteriosis of both the oral and gastric microbiome was possibly associated with early-stage of esophageal squamous cell carcinoma. Larger scale studies and experimental animal models are urgently needed to confirm the possible role of microbiome dysbacteriosis in the pathogenesis of esophageal cancer, especially at an early stage. Also, a comprehensive prediction model that combines both microbial biomarkers and other esophageal cancer risk factors should be further established to increase the accuracy of the screening strategy for early esophageal cancer.

### Electronic supplementary material

Below is the link to the electronic supplementary material.

Supplementary Material 1

Supplementary Material 2

## Data Availability

All the amplicon raw data have been submitted to the Sequence Read Archive (SRA) in NCBI (Archive number: PRJNA961904). In addition, all data from this study can be obtained from the corresponding author upon reasonable request.
